# Laparoscopic versus Open Surgery for Hepatocellular Carcinoma: A Meta-Analysis of High-Quality Case-Matched Studies

**DOI:** 10.1155/2018/1746895

**Published:** 2018-03-01

**Authors:** Ke Chen, Yu Pan, Bin Zhang, Xiao-long Liu, Hendi Maher, Xue-yong Zheng

**Affiliations:** ^1^Department of General Surgery, Sir Run Run Shaw Hospital, School of Medicine, Zhejiang University, 3 East Qingchun Road, Hangzhou, Zhejiang 310016, China; ^2^School of Medicine, Zhejiang University, 866 Yuhangtang Road, Hangzhou, Zhejiang 310058, China

## Abstract

**Objective:**

To present a meta-analysis of high-quality case-matched studies comparing laparoscopic (LH) and open hepatectomy (OH) for hepatocellular carcinoma (HCC).

**Methods:**

Studies published up to September 2017 comparing LH and OH for HCC were identified. Selection of high-quality, nonrandomized comparative studies (NRCTs) with case-matched design was based on a validated tool (Methodological Index for Nonrandomized Studies) since no randomized controlled trials (RCTs) were published. Morbidity, mortality, operation time, blood loss, hospital stay, margin distance, recurrence, and survival outcomes were compared. Subgroup analyses were carried out according to the surgical extension (minor or major hepatectomy).

**Results:**

Twenty studies with a total of 830 patients (388 in LH and 442 in OH) were identified. For short-term surgical outcomes, LH showed less morbidity (RR = 0.55; 95% CI, 0.47~0.65; *P* < 0.01), less mortality (RR = 0.43; 95% CI, 0.18~1.00; *P* = 0.05), less blood loss (WMD = −93.21 ml, 95% CI, −157.33~−29.09 ml; *P* < 0.01), shorter hospital stay (WMD = −2.86, 95% CI, −3.63~−2.08; *P* < 0.01), and comparable operation time (WMD = 9.15 min; 95% CI: −7.61~25.90, *P* = 0.28). As to oncological outcomes, 5-year overall survival rate was slightly better in LH than OH (HR = 0.66, 95% CI: 0.52~0.84, *P* < 0.01), whereas the 5-year disease-free survival rate was comparable between two groups (HR = 0.88, 95% CI: 0.74~1.06, *P* = 0.18).

**Conclusion:**

This meta-analysis has highlighted that LH can be safely performed in selective patients and improves surgical outcomes as compared to OH. Given the limitations of study design, especially the limited cases of major hepatectomy, methodologically high-quality comparative studies are needed for further evaluation.

## 1. Introduction

Although the incidence of hepatocellular carcinoma (HCC) has decreased, HCC is still the fifth most common malignancy and the third leading cause of cancer-related death worldwide [1]. Since laparoscopic hepatectomy (LH) was first reported in 1996 [2, 3], this treatment has been considered a landmark development in the progress of surgical treatment. However, the majority of HCC patients usually have cirrhosis and hypohepatia. Because of this, hepatectomy increases the risk of developing significant postoperative complications including ascites, hepatic failure, encephalopathy, and portal vein thrombosis [4]. There are some controversial aspects of LH for HCC including complications, postoperative recovery, and long-term survival outcomes.

During the last 6 years, a number of meta-analyses that compare LH with open hepatectomy (OH) for HCC have been published [5–8]. Although randomized controlled trials (RCTs) are the most ideal tools for meta-analysis, no RCTs on this topic have been yet conducted. These meta-analyses included the available nonrandomized comparative studies (NRCTs) to overcome the paucity of RCTs. Therefore unreliable results and little strong evidence had been presented. On the other hand, there was evidence that estimates derived from high-quality NRCTs may be similar to those derived from RCTs [9]. Also, when comparing surgical procedures, pooling of high-quality NRCTs could be as accurate as pooling of RCTs [10]. In addition, several comparative studies on this topic have been published in the last 3 years and none of the published meta-analyses included studies published after 2013. Therefore, we performed an updated meta-analysis evaluating all of the available high-quality published trials to compare LH with OH for HCC.

## 2. Methods

### 2.1. Systematic Literature Search

Systematic searches of PubMed, Embase, Cochrane Library, and Web of Science were performed to identify articles published up to September 2017. Searches included the terms “laparoscopic,” “minimally invasive,” “hepatectomy,” “liver resection,” “hepatocellular carcinoma,” and “HCC”. All eligible studies in English were retrieved, and their bibliographies were checked for potential relevant publications.

### 2.2. Eligibility Criteria and Quality Assessment

In order to reduce bias, our meta-analysis synthesized the existing observational studies while strictly limiting inclusion and exclusion criteria. First of all, papers containing any of the following were excluded: (1) studies that included malignant lesions other than HCC, (2) studies focusing on recurrent HCC, (3) studies that included cases of robotic-assisted hepatectomy. Secondly, only studies designed with case-matched analysis were further evaluated and nonmatched studies were excluded. Then, the methodological quality of the eligible nonrandomized comparative studies (NRCTs) was assessed by the Methodological Index for Nonrandomized Studies (MINORS) [11]. In total, 8 items were evaluated, with a maximum score of 16 points. Studies with 12 or more points were considered of high quality and were included in the meta-analysis. Those with less than 12 points were excluded. Besides, if there was overlap between authors or centers, only the higher-quality or more recent literature was selected.

### 2.3. Data Extraction and Quality Assessment

Two researchers evaluated all the titles and abstracts. Then they assessed the selected full-text articles for eligibility. This work was then reevaluated and confirmed by a senior researcher. The measured outcomes of all eligible publications can be divided into two categories: ① short-term outcomes (operation time, estimated blood loss, transfusion, length of hospital stay, morbidity, and mortality); ② oncological outcomes (tumor size, margin distance, R0 resection, recurrence, and survival). The postoperative morbidity was cataloged according to the Clavien-Dindo classification. Minor complication refers to Grade I and Grade II complications, and major complication includes Grade III to V complications.

### 2.4. Subgroup Analysis

Because the different levels of hepatectomy can lead to different outcomes, and major hepatectomy is a technically dependent and time-consuming procedure, subgroup analyses were carried out according to surgical extensions. Included studies were assigned to 3 subgroups: minor hepatectomy, mixed hepatectomy, and major hepatectomy.

### 2.5. Statistical Analysis

The risk ratio (RR) was utilized to analyze the dichotomous variables, and the weighted mean difference (WMD) was utilized to assess the continuous variables. If the study provided medians and ranges instead of means and standard deviations (SDs), we estimated the means and SDs as described by Hozo et al. [12]. Heterogeneity was evaluated by Cochran's *Q* statistic and Higgins *I*^2^ statistic [13]. If data was not significantly heterogeneous (*P* > 0.05 or *I*^2^ < 50%), the pooled effects were calculated using a fixed model. Otherwise, the pooled effects were calculated using a random model. The hazard ratios (HRs) of a 5-year overall survival rate (OS) and a 5-year disease-free survival rate (DFS) were used with a generic inverse variance meta-analysis. The log HR and its SE were estimated using the method introduced by Tierney et al. [14]. According to the overall morbidity, potential publication bias was determined by carrying out an informal visual inspection of funnel plots. A two-tailed value of *P* < 0.05 was considered significant. All statistical tests were performed with Review Manager version 5.1 (The Cochrane Collaboration, Oxford, England).

## 3. Results

### 3.1. Search Results and Baseline Characteristics

**Figure 1:**
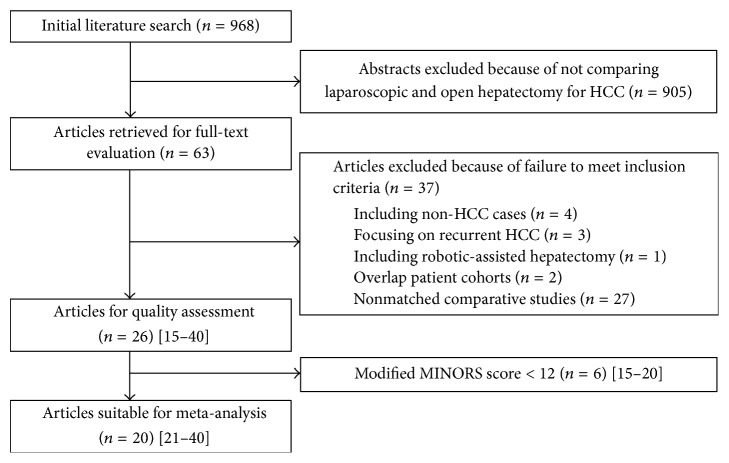
Flow chart of literature search strategies.

The last search was performed on September 20, 2017. A total of 968 potential published articles were initially identified from the search. Of these, 63 articles were selected based on their titles and abstracts, and a full examination of the texts was performed. Further 37 papers were excluded, after being read thoroughly, due to (1) including non-HCC cases (*n* = 4), (2) focusing on recurrent HCC (*n* = 3), (3) robot-assisted hepatectomy (*n* = 1), (4) overlap patient cohorts (*n* = 2), or (5) nonmatched comparative studies (*n* = 27). Then 26 studies were selected for quality assessment, and 6 studies were excluded by a modified MINORS score < 12 [15–20]. Finally, 20 studies were selected for final meta-analysis [21–40]. A flow chart of the search strategies, which contains reasons for excluding studies, is elucidated in Figure 1. The details of the selection process, which included the references of excluded studies and the MINORS assessments of low-quality studies, could be found in Supplementary Materials (available here).

### 3.2. Study Characteristics

A total of 830 patients were included in the analysis with 388 undergoing LH (46.8%) and 442 undergoing OH (53.2%). The characteristics of these included studies are summarized in Table 1. Studies were well matched in terms of age, gender, ASA classification, body mass index (BMI), tumor size, and surgical extension. Eight studies reported only minor hepatectomy, and three studies focused on major hepatectomy, whereas the remaining nine studies included both minor and major hepatectomy. The majority of studies graded morbidity according to the Clavien-Dindo classification, with the study by Lee et al. being the only exception [24]. The assessments of the NRCTs are illustrated in Table 2. Each trial received more than 12 points (the maximum possible score is 16) and was considered to be of the highest quality (see Supplementary Materials).

### 3.3. Meta-Analysis of Short-Term Outcomes

#### 3.3.1. Operation Time

Operative time was reported in all studies [21–40]. Statistically significant between-study heterogeneity was identified in all subgroups (*P* < 0.01, *I*^2^ = 87.2%). There was no significant difference between the groups' operation times (Table 3). However, in the subgroup of major hepatectomy, the overall effect size of the mean operation time was significantly longer in LH than that in OH (WMD = 77.93 min, 95% CI: 40.45~115.41, *P* < 0.01).

#### 3.3.2. Intraoperative Blood Loss

Blood loss was available from 17 studies [21, 22, 24–34, 36, 37, 39, 40]. Statistically significant between-study heterogeneity was identified in all subgroups (*P* < 0.01, *I*^2^ = 88.1%). The pooled results showed that LH was associated with less blood loss than OH (Table 3). However, in the subgroup of major hepatectomy, there was no significant difference between groups (WMD = 3.75 ml, 95% CI: −60.16~67.65, *P* = 0.88).

#### 3.3.3. Blood Transfusion

Fourteen studies recorded perioperative blood transfusion [21–27, 29, 30, 33–35, 37, 39]. There was no evidence of heterogeneity between subgroups (*P* = 0.81, *I*^2^ = 0%). Although none of the subgroups reached a significant difference, the overall pooled data indicated that transfusion rates were lower in LH (RR = 0.73, 95% CI: 0.55~0.96,  *P* = 0.03) (Table 3).

#### 3.3.4. Duration of Hospital Stay

The length of hospital stays was pooled for all studies [21–40]. Although statistically significant between-study heterogeneity was identified in each subgroup, there was no evidence of heterogeneity between subgroups (*P* = 0.99, *I*^2^ = 0%). Hospital stays in LH group were shorter than those in OH group (WMD = −2.86 d, 95% CI: −3.63~−2.08, *P* < 0.01) (Table 3).

#### 3.3.5. Morbidity

**Figure 2:**
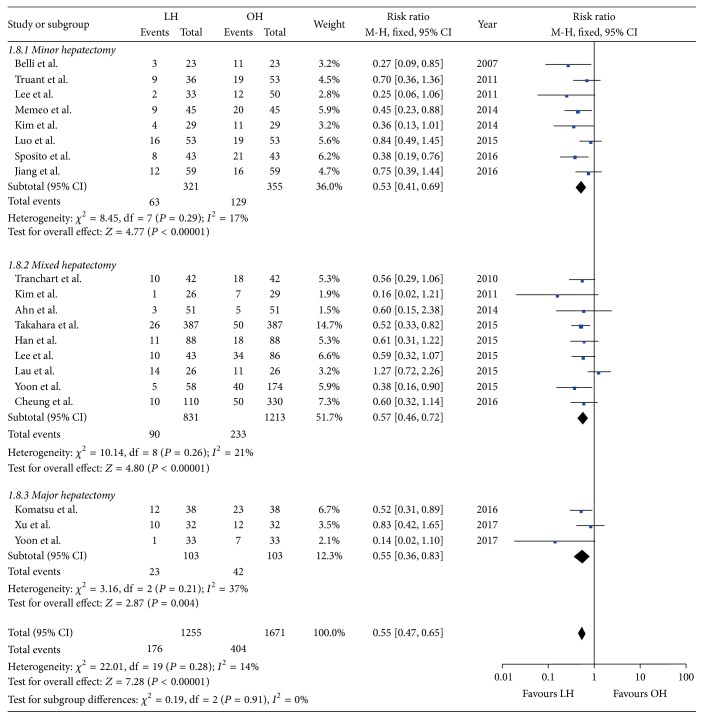
Forest plot of overall morbidity.

All studies reported their overall complication rates [21–40]. Because there was no statistical evidence of heterogeneity, the effect sizes of all subgroups were synthesized to generate the overall effect size (*P* = 0.91, *I*^2^ = 0%) (Table 3) (Figure 2). The postoperative morbidity rates were 14.0% (176/1255) in LH and 24.2% (404/1671) in OH. In addition the pooled data showed that LH significantly reduced postoperative complications (RR = 0.55; 95% CI, 0.47~0.65; *P* < 0.01) (Table 3). Moreover, each subgroup also revealed reduced overall morbidity in the LH group (Table 3).

**Figure 3:**
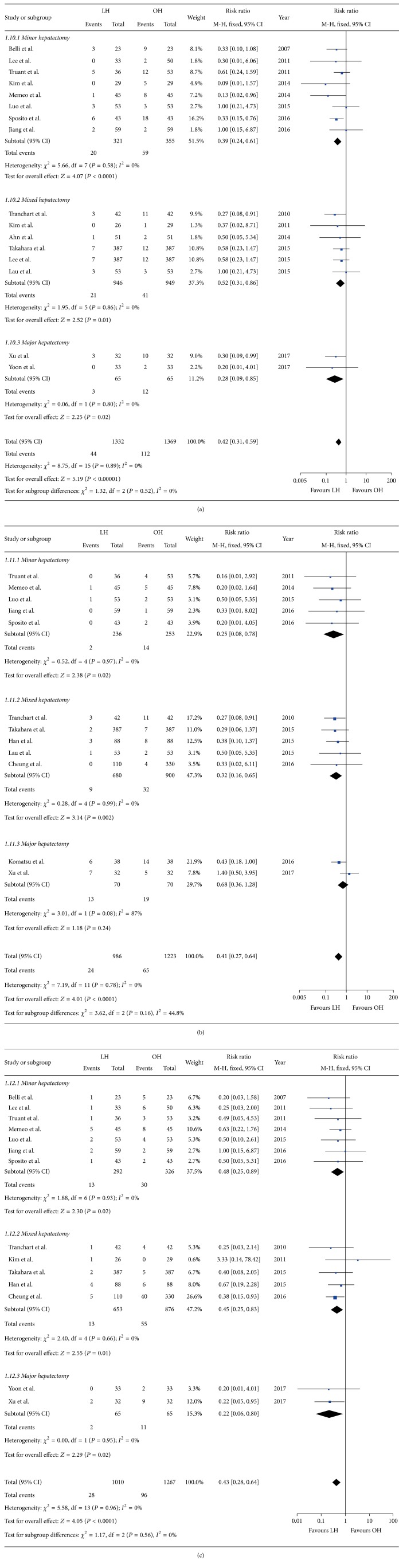
Forest plot of specific complications: (a) ascites, (b) liver failure, (c) respiratory complications.

Eighteen studies recorded severe complications [21, 22, 25–40]. Similar to overall morbidity, the results showed that patients in the LH group suffered less severe complications (Table 3). We identified specified complications of ascites, liver failure, and the respiratory system. The results implied that postoperative ascites in patients, regardless of whether they underwent minor or major hepatectomy, was less in LH than in OH (RR = 0.42; 95% CI, 0.31~0.59; *P* < 0.01) (Figure 3(a)). Studies that recorded postoperative liver failure reported a lower incidence of liver failure in LH than in OH with one exception by Xu et al. [39]. The overall pooled data revealed that patients in the LH group were less likely to suffer liver failure than those in the OH group (RR = 0.41; 95% CI, 0.27~0.64; *P* < 0.01) (Figure 3(b)). LH was also associated with a significant reduction in respiratory complications regardless of different surgical extension (RR = 0.43, 95% CI: 0.28~0.64, *P* < 0.01) (Figure 3(c)).

#### 3.3.6. Mortality

Nine studies recorded cases of postoperative death [21, 22, 25, 28–30, 33, 35, 39]. There was no evidence of heterogeneity between subgroups (*P* = 0.97, *I*^2^ = 0%). These studies showed very low incidences of mortality. However, the overall pooled data indicated a more reduced postoperative mortality in LH than that in OH (RR = 0.43; 95% CI, 0.18~1.00; *P* = 0.05) (Table 3).

### 3.4. Meta-Analysis of Oncological Outcomes

#### 3.4.1. Tumor Size

Only one study did not report tumor size [30]. There was trifling heterogeneity between subgroups, mainly due to the major hepatectomy subgroup (*P* = 0.35, *I*^2^ = 4.2%) (Table 3). Meta-analysis showed that the tumor size of OH was longer than that of LH with a marginal difference (WMD = −0.19 cm; 95% CI: −0.41~−0.03, *P* = 0.09), which was mainly due to smaller tumors in LH than those in OH in the major hepatectomy subgroup (Table 3).

#### 3.4.2. Margin Distance

Only 11 studies mentioned the distance of the tumor margin [22, 24–29, 31, 34, 38, 40]. Although statistical significant between-study heterogeneity was identified in each subgroup, there was no evidence of heterogeneity between subgroups (*P* = 0.63, *I*^2^ = 0%). On pooling the results, the margin distance was longer in the LH group than that in the OH group (WMD = 2.61 cm; 95% CI: 1.06~4.17, *P* < 0.01) (Table 3).

#### 3.4.3. R0 Resection

The R0 resection was reported in 14 studies [21, 23, 24, 27, 29–36, 38, 39]. There was no obvious heterogeneity (*P* = 0.18, *I*^2^ = 41.8%). The pooled estimate for margin distance indicated comparative outcomes between groups (RR = 1.01, 95% CI: 0.99~1.02, *P* = 0.37) (Table 3).

#### 3.4.4. Overall Survival Rate and Disease-Free Survival Rate

**Figure 4:**
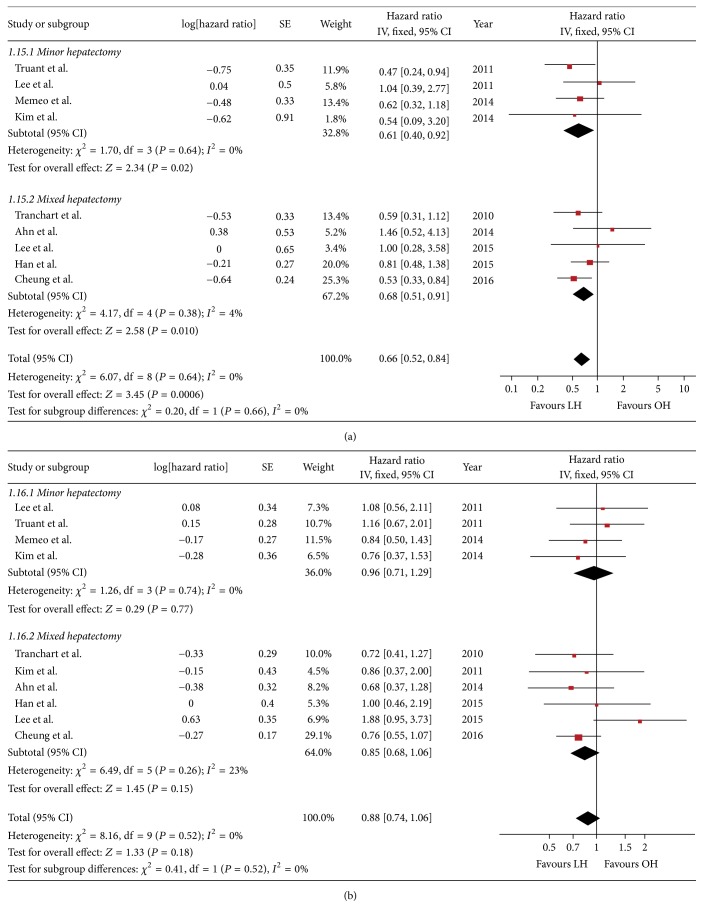
Forest plot of survival rate: (a) 5-year OS, (b) 5-year DFS.

Summary of follow-up time, recurrence, and long-term survival rates is listed in Table 4. Nineteen studies reported the detailed long-term outcomes. Among them, the data for 5-year OS rates can be extracted from nine studies and the data for 5-year DFS rates can be extracted from ten studies. The follow-up periods in six studies were less than five years. The survival data of three studies cannot be extracted due to a technical problem with figures. Unfortunately, none of the three major hepatectomy studies can be included in our survival analysis [37, 39, 40]. In all, the pooled 5-year OS rate was slightly better in LH than in OH (HR = 0.66, 95% CI: 0.52~0.84, *P* < 0.01) (Figure 4(a)). The 5-year DFS rate was comparable between groups (HR = 0.88, 95% CI: 0.74~1.06, *P* = 0.18) (Figure 4(b)).

#### 3.4.5. Publication Bias

**Figure 5:**
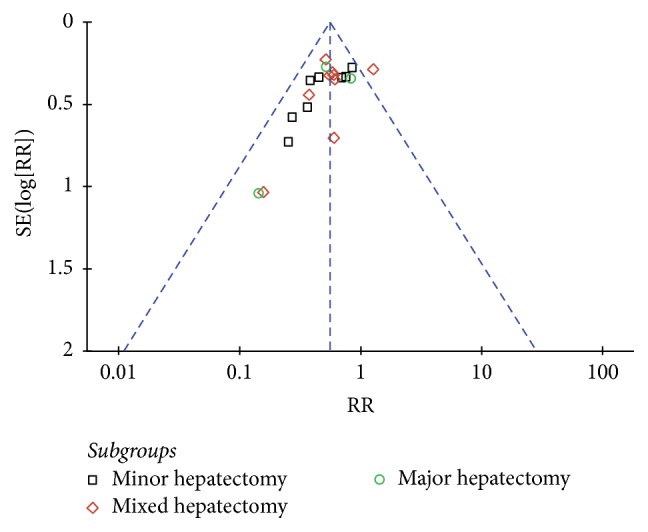
Funnel plot of the overall postoperative morbidity.

The study by Lau et al. was outside the funnel [30], and the remaining representative plots were distributed symmetrically. We believed such publication bias was acceptable in the studies (Figure 5).

## 4. Discussion

This meta-analysis selected and summarized high-quality literature that compared the short- and long-term outcomes of LH and OH for the treatment of HCC. All of the studies had case-matched design and were of high quality according to the modified MINORS scale. For short-term surgical outcomes, LH exhibited advantages in terms of blood loss, hospital stay, overall postoperative morbidity, and mortality, whereas no statistically significant differences were identified regarding operation time. As for oncological outcomes, R0 and survival rates of LH were also not inferior to OH.

To date, there have been several meta-analyses comparing LH to OH for HCC [5–8]. The results have demonstrated that LH is comparable to OH regarding the operation time and postoperative mortality and is associated with less blood loss, as well as a shorter hospital stay (Table 5). Previous meta-analyses included all available research [5, 6, 8] but had some limitations. Pooling of low-quality studies could undermine the strength of results, whereas selectively pooling high-quality NRCTs could strengthen the power of results [10]. Patients' characteristics and surgical extension have a major impact on the surgical outcomes of hepatectomy. Previous meta-analyses pooled studies, which did not balance the combined factors of tumor size, location, the severity of cirrhosis, and other underlying liver diseases between LH and OH. These factors would have influenced the decision of surgeons and patients and further influenced the major factors of both short- and long-term outcomes. In addition, previous meta-analyses studied LH confined to minor resection. With the accumulation of surgical techniques, major resection of LH has become more commonly performed, but various efficacy and safety concerns for the procedure are warranted. Furthermore, since the publication of previous meta-analyses, several notable clinical observational studies have become available and some of them are from China, where HCC has the highest prevalence in the world [8, 19]. Therefore, our comprehensive meta-analysis will contribute to a more systematic and objective evaluation of the safety and HCC treatment of LH.

Several previous studies have demonstrated that LH can be feasible and beneficial for minor resections or nonanatomical resections of peripheral HCC. This is in accordance with our study that showed minor resection of LH with similar operation time and less blood loss than OH. However, minor hepatectomy is insufficient for large lesions or those located in posterosuperior liver segments to ensure an adequate resection margin and eliminate intrahepatic recurrence. Major hepatectomy is more frequently performed with a curative intent for multifocal or large size HCC or those with a high propensity to invade the portal vein branches [41–43]. Laparoscopic major hepatectomy is, because of the same steps and principles used in laparotomy, technically demanding. Mobilization of a heavy as well as fragile organ, excisions of bulky parenchyma, and major vascular dissection with its associated risk of major vessel injury are all considered risky under laparoscopy. As expected, the present study revealed longer operation times in laparoscopic major hepatectomy. Furthermore, unlike minor resections, the blood loss of laparoscopic major hepatectomy was not superior to its open counterpart.

Patients with HCC and concurrent cirrhosis tend to have higher incidences of postoperative complications and of greater severity. Therefore, the decreased complications in the LH group should be our most striking finding. In detail, postoperative ascites and liver failure tend to decrease in LH. Postoperative decompensation after hepatectomy occurs more frequently in patients with liver cirrhosis or portal hypertension, even for limited resections. The minimization of surgical incision and the subsequent preservation of abdominal wall circulation and lymphatic flow can explain fewer ascites and liver failure in LH. Moreover, a small incision limits the evacuation of ascites through the wall and decreases the risk of infection, thus facilitating wound healing. Laparoscopic surgery also decreases the manipulation of abdominal organs and exposure of bowels, which will also contribute to reduced ascites. Since refractory ascites and progressive liver insufficiency are major causes of severe postoperative morbidities, reduced severe postoperative morbidities and mortality could be expected. Major surgery was often thought to be unsuitable for those with severely impaired pulmonary function due to a higher risk of postoperative respiratory complications. Hepatectomy involving multiple systems, especially the water and electrolyte balance, is a major risk factor for medical complications. It was observed from the reviewed studies that respiratory complications were the most common medical complications, mainly pulmonary infection, followed by cardiovascular complications. Improved preservation of liver functions in LH maintains enough albumin synthesis and decreases the pleural effusion. The pain caused by large incisions, as well as the use of tension sutures and abdominal bandages after laparotomy, can make it difficult for patients to cough. Earlier postoperative ambulation in the laparoscopic group also helped to reduce respiratory complications and promote the postoperative recovery of gastrointestinal function. In accordance with other laparoscopic surgeries, LH achieved enhanced postoperative recovery. The postoperative hospitalization of LH decreases by more than two days. This can be explained by the milder surgical trauma of LH and subsequent faster bowel recovery. Less postoperative morbidities also contribute to shorter length of hospitalization.

The oncologic results of LH for HCC remain a matter of debate. Adequate surgical margins independently improve the long-term oncological outcomes. Our analysis showed that LH could achieve enough surgical margins (more than 2 cm) as OH. The 5-year OS and DFS also showed that LH was comparable to OH. However, the results warrant prudent interpretation because of the discrepancies among the pooled studies, such as tumor size, tumor number, and status of the vascular invasion. Other biases lie in other factors including preoperative TACE and postoperative adjuvant therapies. Unfortunately, none of the three major hepatectomy studies can be included in our survival analysis. Thus, well-designed RCTs, that balance all potential factors, preferably containing major resection are needed to confirm our results.

In the process of our research and manuscript review, two similar articles by Sotiropoulos et al. were published [44, 45], which also had limitations. Examples include pooling the low-quality studies together, failing to evaluate extension on surgical outcomes, and one paper only investigating studies conducted in Europe [45]. Besides, since these studies were published, several clinical observational studies have become available. Therefore, our comprehensive meta-analysis will contribute to a more systematic and objective evaluation of this subject.

The major limitation of this study was that all included studies are NRCTs and of retrospective design. NRCTs have potential biases that limit an unequivocal conclusion, even though we exclusively included the case-matched studies to minimize the selection biases. Another limitation is the lack of studies on laparoscopic major hepatectomy. The analysis was based on only three pooled studies. Little is known about how these results would hold for a larger sample size, which is particularly important as a fair number of patients with HCC are treated with open major hepatectomy. In addition, data from several studies are extracted using the methods reported by Hozo et al. and Tierney et al., which are not completely accurate and result in bias. Moreover, it is quite possible that surgical teams undertaking research and publishing their results are more experienced and more skillful than others. Publication bias was inevitable since one plot was outside the funnel. The bias would be overcome only with the collection of more reports.

## 5. Conclusions

This meta-analysis has highlighted that LH can be safely performed in select patients and improves surgical outcomes when compared to OH. The data indicate that laparoscopic minor hepatectomy is acceptable with less blood loss, less postoperative morbidity, shorter hospitalization, and comparable operation times and oncological outcomes. The role of laparoscopic major hepatectomy is promising in terms of decreasing postoperative morbidity and recovery, but the technique also has drawbacks in prolonged operation time. Given the heterogeneity of the patient groups, the limitations of study design, and the small sample size, it is likely that patients have potential to benefit from LH, but further well-designed studies are needed to accurately select them.

## Figures and Tables

**Table 1:** Basic information of the included literature.

Author	Region	Year	Study period	Sample size		Matching method	Cirrhosis (%)		Surgical extension	Conversion (%)	Clavien-Dindo
				LH	OH		LH	OH			
Belli et al.	Italy	2007	2000–2004	23	23	CCM	100	100	Minor	4.3	Yes
Tranchart et al.	France	2010	1999–2008	42	42	CCM	73.8	81	Mixed	4.8	Yes
Kim et al.	Korea	2011	2005–2009	26	29	CCM	92.3	86.2	Mixed	E	Yes
Truant et al.	France	2011	2002–2009	36	53	CCM	100	100	Minor	19.4	Yes
Lee et al.	Hong Kong	2011	2004–2010	33	50	CCM	84.8	64	Minor	18.2	NA
Ahn et al.	Korea	2014	2005–2013	51	51	PSM	68.6	66.8	Mixed	9.8	Yes
Kim et al.	Korea	2014	2000–2012	29	29	PSM	62.1	65.5	Minor	E	Yes
Memeo et al.	France	2014	1990–2009	45	45	CCM	100	100	Minor	NA	Yes
Lau et al.	USA	2015	2008–2014	26	26	CCM	80.8	73.1	Mixed	35	Yes
Lee et al.	Canada	2015	2006–2013	43	86	CCM	NA	NA	Mixed	14	Yes
Luo et al.	China	2015	2008–2015	53	53	CCM	100	100	Minor	E	Yes
Takahara et al.	Japan	2015	2000–2010	387	387	PSM	61.7	59.6	Mixed	6.5	Yes
Han et al.	Korea	2015	2004–2013	88	88	PSM	62.5	59.1	Mixed	9.1	Yes
Yoon et al.	Korea	2015	2007–2011	58	174	PSM	NA	NA	Mixed	0	Yes
Cheung et al.	Hong Kong	2016	2002–2015	110	330	PSM	100	100	Mixed	5.5	Yes
Sposito et al.	Italy	2016	2006–2013	43	43	PSM	100	100	Minor	4.7	Yes
Jiang et al.	China	2016	2008–2013	59	59	PSM	100	100	Minor	5.1	Yes
Komatsu et al.	France	2016	2000–2014	38	38	CCM	NA	NA	Major	31.6	Yes
Yoon et al.	Korea	2017	2008–2015	33	33	PSM	100	100	Major	NA	Yes
Xu et al.	China	2017	2015–2017	32	32	PSM	100	100	Major	NA	Yes

CCM: case by case matching; PSM: propensity score matching; E: conversion cases were excluded from the studies; NA: not available.

**Table 2:** Modified MINORS score of all eligible nonrandomized comparative studies.

Author	①	②	③	④	⑤	⑥	⑦	⑧	Score
Belli et al.	2	2	1	2	2	1	2	1	13
Tranchart et al.	2	2	1	1	2	1	2	2	13
Kim et al.	2	1	1	2	2	2	1	1	12
Truant et al.	2	2	1	1	2	2	1	1	12
Lee et al.	2	2	1	1	2	2	1	1	12
Ahn et al.	2	2	1	1	2	2	2	2	14
Kim et al.	2	1	1	2	2	2	1	1	12
Memeo et al.	2	1	1	1	2	1	2	2	12
Lau et al.	2	1	2	1	2	2	2	1	13
Lee et al.	2	1	1	1	2	2	1	2	12
Luo et al.	2	1	1	1	2	2	2	2	13
Takahara et al.	2	1	1	1	2	2	2	2	13
Han et al.	2	1	1	1	2	2	2	2	13
Yoon et al.	2	1	1	1	2	2	1	2	12
Cheung et al.	2	2	1	1	2	2	2	2	14
Sposito et al.	2	2	1	1	2	2	2	2	14
Jiang et al.	2	1	1	1	2	2	2	2	13
Komatsu et al.	2	1	1	1	2	2	2	1	12
Yoon et al.	2	1	2	2	2	2	2	1	14
Xu et al.	2	2	2	1	2	2	2	1	14

① Consecutive patients, ② prospective data collection, ③ reported endpoints, ④ unbiased outcome evaluation, ⑤ appropriate controls, ⑥ contemporary groups, ⑦ groups equivalent, ⑧ sample size.

**Table 3:** Overall outcomes of the meta-analysis.

Outcomes	Studies No.	Sample size		Heterogeneity (*P*, *I*^2^)	Model	Overall effect size	95% CI of overall effect	*P*
		LH	OH					
*Operation time (min)*	20	1255	1671	<0.01, 87%	R	WMD = 9.15	−7.61~25.90	0.28
Minor hepatectomy	8	321	355	<0.01, 78%	R	WMD = 12.04	−5.31~29.39	0.17
Mixed hepatectomy	9	831	1213	<0.01, 83%	R	WMD = −14.28	−40.76~12.21	0.29
Major hepatectomy	3	103	103	0.05, 67%	R	WMD = 77.93	40.45~115.41	**<0.01**
*Blood loss (mL)*	17	1128	1425	<0.01, 92%	R	WMD = −93.21	−157.33~−29.09	**<0.01**
Minor hepatectomy	7	278	312	0.39, 5%	R	WMD = −76.21	−98.41~−54.01	**<0.01**
Mixed hepatectomy	7	747	1010	0.05, 52%	R	WMD = −212.94	−294.57~−131.31	**<0.01**
Major hepatectomy	3	103	103	0.88, 0%	R	WMD = 3.75	−60.16~67.65	0.88
*Transfusion*	14	979	1352	0.90, 0%	F	RR = 0.73	0.55~0.96	**0.03**
Minor hepatectomy	4	121	155	0.49, 0%	F	RR = 0.53	0.19~1.45	0.22
Mixed hepatectomy	8	788	1127	0.86, 0%	F	RR = 0.75	0.55~1.01	0.06
Major hepatectomy	2	70	70	0.28, 15%	F	RR = 0.75	0.17~3.25	0.70
*Hospital stay (days)*	20	1255	1671	<0.01, 80%	R	WMD = −2.86	−3.63~−2.08	**<0.01**
Minor hepatectomy	8	321	355	<0.01, 76%	R	WMD = −2.93	−4.23~−1.63	**<0.01**
Mixed hepatectomy	9	831	1213	0.01, 58%	R	WMD = −2.85	−3.95~−1.76	**<0.01**
Major hepatectomy	3	103	103	0.15, 47%	R	WMD = −2.76	−4.60~−0.92	**<0.01**
*Morbidity*	20	1255	1671	0.28, 14%	F	RR = 0.55	0.47~0.65	**<0.01**
Minor hepatectomy	8	321	355	0.29, 17%	F	RR = 0.53	0.41~0.69	**<0.01**
Mixed hepatectomy	9	831	1213	0.26, 21%	F	RR = 0.57	0.46~0.72	**<0.01**
Major hepatectomy	3	103	103	0.21, 37%	F	RR = 0.55	0.36~0.83	**<0.01**
*Severe complications*	18	1196	1476	0.88, 0%	F	RR = 0.51	0.39~0.68	**<0.01**
Minor hepatectomy	7	288	305	0.96, 0%	F	RR = 0.48	0.24~0.96	**0.04**
Mixed hepatectomy	8	805	1068	0.44, 0%	F	RR = 0.54	0.38~0.76	**<0.01**
Major hepatectomy	3	103	103	0.35, 6%	F	RR = 0.42	0.18~1.00	**0.05**
*Mortality*	9	789	1026	0.88, 0	F	RR = 0.43	0.18~1.00	**0.05**
Minor hepatectomy	3	104	121	0.34, 7	F	RR = 0.39	0.09~1.68	0.21
Mixed hepatectomy	5	653	873	0.84, 0	F	RR = 0.46	0.15~1.43	0.18
Major hepatectomy	1	32	32	Not applicable	F	RR = 0.33	0.01~7.89	0.50
*Tumor size (cm)*	19	1229	1645	<0.01, 57%	R	WMD = −0.19	−0.41~0.03	0.09
Minor hepatectomy	8	321	355	0.76, 0%	R	WMD = −0.07	−0.26~0.12	0.48
Mixed hepatectomy	8	805	1187	0.50, 0%	R	WMD = −0.09	−0.25~0.07	0.28
Major hepatectomy	3	103	103	<0.01, 92%	R	WMD = −1.77	−4.06~0.53	0.13
*Margin distance (cm)*	11	501	694	0.14, 47%	R	WMD = 2.61	1.06~4.17	**<0.01**
Minor hepatectomy	5	186	220	0.06, 56%	R	WMD = 2.16	0.15~4.17	**0.03**
Mixed hepatectomy	5	282	441	0.15, 41%	R	WMD = 3.20	0.41~5.99	**0.02**
Major hepatectomy	1	33	33	Not applicable	R	WMD = 5.90	−2.69~14.49	0.18
*R0 resection*	14	1010	1409	0.70, 0%	F	RR = 1.01	0.99~1.02	0.37
Minor hepatectomy	6	240	257	0.80, 0%	F	RR = 0.98	0.95~1.01	0.23
Mixed hepatectomy	7	738	1120	0.47, 0%	F	RR = 1.01	1.00~1.03	0.13
Major hepatectomy	1	32	32	Not applicable	F	RR = 1.03	0.93~1.15	0.56

WMD: weighted mean difference; RR: risk ratio; F: fixed; R: random.

**Table 4:** Summary of recurrence and long-term survival.

Author	Group	Follow-up	R	Survival (time: month; rate: %)
Tranchart et al.	LH	29.7	10	1, 3, 5 y-DFS: 81.6, 60.9, 45.6; 1, 3, 5 y-OS: 93.1, 74.4, 59.5.
	OH	24.6	12	1, 3, 5 y-DFS: 70.2, 54.3, 37.2; 1, 3, 5 y-OS: 81.8, 73, 47.4.
Kim et al.	LH	21.8	7	MDFS: 13.4; 1 y-DFS: 84.6.
	OH	24.8	10	MDFS: 14.6; 1 y-DFS: 82.8.
Truant et al.	LH	35.7	16	5 y-DFS: 35.5; 5 y-OS: 70.
	OH		23	5 y-DFS: 33.6; 5 y-OS: 46.
Lee et al.	LH	35.4	15	1, 3, 5 y-DFS: 78.8, 51, 45.3; 1, 3, 5 y-OS: 86.9, 81.8, 76.0.
	OH	28.5	19	1, 3, 5 y-DFS: 69.2, 55.9, 55.9; 1, 3, 5 y-OS: 98, 80.6, 76.1.
Ahn et al.	LH	38.6	12	5 y-DFS: 67.8; 5 y-OS: 80.1.
	OH	52.3	21	5 y-DFS: 54.8; 5 y-OS: 85.7.
Kim et al.	LH	47.9	11	MDFS: 15.4; MOS: 47.9; 1, 3, 5 y-DFS: 81.1, 61.7, 54.0; 1, 3, 5 y-OS: 100, 100, 92.2.
	OH	59.5	16	MDFS: 32.6; MOS: 59.5; 1, 3, 5 y-DFS: 78.6, 60.9, 40.1; 1, 3, 5 y-OS: 96.5, 92.2, 87.7.
Memeo et al.	LH	NR	25	1, 5, 10 y-DFS: 80, 19, 0; 1, 5, 10 y-OS: 88, 59, 12.
	OH	NR	28	1, 5, 10 y-DFS: 60, 23, 9; 1, 5, 10 y-OS: 63, 44, 22.
Lee et al.	LH	22.7	NR	1, 3, 5 y-DFS: 60.5, 53.5, 53.5; 1, 3, 5 y-OS: 95.3, 89.7, 89.7.
	OH	44.4	NR	1, 3, 5 y-DFS: 81.5, 66.7, 58.6; 1, 3, 5 y-OS: 93.9, 89.5, 87.3.
Luo et al.	LH	35	20	MDFS: 21.
	OH	37	24	MDFS: 18.
Takahara et al.	LH	46.7	NR	1, 3, 5 y-DFS: 83.7, 58.3, 40.7; 1, 3, 5 y-OS: 95.8, 86.2, 76.8.
	OH	51.7	NR	1, 3, 5 y-DFS: 79.6, 50.4, 39.3; 1, 3, 5 y-OS: 95.8, 84.0, 70.9.
Han et al.	LH	44.0	43	1, 3, 5 y-DFS: 69.7, 52.0, 44.2; 1, 3, 5 y-OS: 91.6, 87.5, 76.4.
	OH	48.7	46	1, 3, 5 y-DFS: 74.7, 49.5, 41.2; 1, 3, 5 y-OS: 93.1, 87.8, 73.2.
Yoon et al.	LH	NR	16	1, 2, 3, 4 y-DFS: 82.0, 63.0, 56.0, 56.0; 1, 2, 3, 4 y-OS: 95.0, 92.0, 86.0, 86.0.
	OH	NR	31	1, 2, 3, 4 y-DFS: 88.0, 79.0, 62.0, 62.0; 1, 2, 3, 4 y-OS: 98.0, 93.0, 84.0, 68.0.
Cheung et al.	LH	34.6	36	MDFS: 66.4; MOS: 136; 1, 3, 5 y-DFS: 87.7, 65.8, 52.2; 1, 3, 5 y-OS: 98.9, 89.8, 83.7.
	OH	46.6	160	MDFS: 52.4; MOS: 120; 1, 3, 5 y-DFS: 75.2, 56.3, 47.9; 1, 3, 5 y-OS: 94, 79.3, and 67.4.
Sposito et al.	LH	39.3	NR	MDFS: 25.5; MOS: 48.8; 3, 5 y-DFS: 41, 25; 3, 5 y-OS: 75, 38.
	OH	44.5	NR	MDFS: 31.7; MOS: 57.8; 3, 5 y-DFS: 44, 11; 3, 5 y-OS: 79, 46.
Jiang et al.	LH	NR	26	MDFS: 17; 5 y-DFS: 44.
	OH	NR	30	MDFS: 15; 5 y-DFS: 40.
Komatsu et al.	LH	24.7	NR	3 y-DFS: 50.3; 3 y-OS: 73.4.
	OH		NR	3 y-DFS: 29.7; 3 y-OS: 69.2.
Yoon et al.	LH	NR	NR	2 y-DFS: 85.1; 2 y-OS: 100.
	OH	NR	NR	2 y-DFS: 83.9; 2 y-OS: 88.8.
Xu et al.	LH	13.8	NR	1, 2 y-DFS: 95.5, 72.9; 1, 2 y-OS: 100, 85.7.
	OH		NR	1, 2 y-DFS: 93.5, 81.5; 1, 2 y-OS: 96.3, 86.7.

Follow-up was shown as median month; R: recurrence; DFS: disease-free survival rate; OS: overall survival rate; MDFS: median disease-free survival time; MOS: median overall survival time; y: year; NR: not reported.

**Table 5:** Previous meta-analyses comparing LH to OH for HCC.

Variables	Zhou	Li	Xiong	Yin
Year	2011	2012	2012	2013
Included studies	10	10	9	15
Total LH numbers	213	244	234	485
Surgical extension	Minor resection	Minor resection	Minor resection	Minor resection
Operation time	NS	NS	NS	NS
Blood loss	Favor LH	Favor LH	Favor LH	Favor LH
Overall morbidity	Favor LH	Favor LH	N/A	Favor LH
Severe complications	N/A	N/A	N/A	N/A
ascites	N/A	N/A	Favor LH	N/A
Liver failure	NS	N/A	Favor LH	N/A
Respiratory complications	NS	N/A	NS	N/A
Mortality	NS	N/A	NS	N/A
Hospital stay	Favor LH	Favor LH	Favor LH	Favor LH
Tumor size	N/A	N/A	N/A	N/A
Margin distance	NS	NS	N/A	NS
R0 resection	N/A	NS	NS	NS
Survival	N/A	N/A	N/A	NS

NS: not significant, N/A: not available.

## Abbreviations

LH: Laparoscopic hepatectomy
OH: Open hepatectomy
HCC: Hepatocellular carcinoma
NRCT: Nonrandomized comparative study
RCT: Randomized controlled trial
MINORS: Methodological Index for Nonrandomized Studies
RR: Risk ratio
WMD: Weighted mean difference
SD: Standard deviation
OS: Overall survival rate
DFS: Disease-free survival rate
HR: Hazard ratio.

## Supplementary Materials

Supplementary Materials Table of modified MINORS score of studies with score < 12.
