# Supplementary material for: Laparoscopic versus Open Surgery for Hepatocellular Carcinoma: A Meta-Analysis of High-Quality Case-Matched Studies
Source: Can J Gastroenterol Hepatol. 2018 Mar 1;2018:1746895. doi: 10.1155/2018/1746895 (PMC5852873; doi:10.1155/2018/1746895)
Supplement: Supplementary Materials — Table of modified MINORS score of studies with score < 12. [file 1746895.f1.docx]

**Reasons of excluded articles and references**

Including non-HCC cases (n=4) [[1-4](#_ENREF_1)]

Focusing on recurrent HCC (n=3) [[5-7](#_ENREF_5)]

Including robotic assisted hepatectomy (n=1) [[8](#_ENREF_8)]

Overlap patient cohorts (n=2) [[9](#_ENREF_9), [10](#_ENREF_10)]

Non-matched comparative studies (n=27) [[11-37](#_ENREF_11)]

Modified MINORS Index score < 12 (n=6) [[38-43](#_ENREF_38)]

**Table** Modiﬁed MINORS Score of Studies with score < 12.

| **Author** | **①** | **②** | **③** | **④** | **⑤** | **⑥** | **⑦** | **⑧** | **Score** |
| --- | --- | --- | --- | --- | --- | --- | --- | --- | --- |
| Laurent[[38](#_ENREF_38)] | 2 | 2 | 1 | 1 | 2 | 1 | 1 | 0 | 10 |
| Lai[[39](#_ENREF_39)] | 2 | 2 | 1 | 1 | 2 | 1 | 0 | 1 | 10 |
| Sarpel[[40](#_ENREF_40)] | 2 | 2 | 1 | 1 | 2 | 1 | 0 | 1 | 10 |
| Aldrighetti[[41](#_ENREF_41)] | 2 | 2 | 1 | 1 | 2 | 1 | 2 | 0 | 11 |
| Hu[[42](#_ENREF_42)] | 2 | 1 | 1 | 1 | 2 | 2 | 1 | 1 | 11 |
| Meguro[[43](#_ENREF_43)] | 2 | 1 | 1 | 1 | 2 | 2 | 1 | 1 | 11 |

①Consecutive Patients, ②Prospective Data Collection, ③Reported Endpoints, ④Unbiased outcome evaluation, ⑤Appropriate controls, ⑥Contemporary Groups, ⑦Groups Equivalent, ⑧Sample Size.

**References**

1. Lee KF, Cheung YS, Chong CN, Tsang YY, Ng WW, Ling E, Wong J, Lai PB. Laparoscopic versus open hepatectomy for liver tumours: a case control study. Hong Kong Med J. 2007; 13: 442-8. doi:

2. Chan AC, Poon RT, Cheung TT, Chok KS, Dai WC, Chan SC, Lo CM. Laparoscopic versus open liver resection for elderly patients with malignant liver tumors: a single-center experience. J Gastroenterol Hepatol. 2014; 29: 1279-83. doi: 10.1111/jgh.12539.

3. Hirokawa F, Hayashi M, Miyamoto Y, Asakuma M, Shimizu T, Komeda K, Inoue Y, Uchiyama K. Short- and long-term outcomes of laparoscopic versus open hepatectomy for small malignant liver tumors: a single-center experience. Surg Endosc. 2015; 29: 458-65. doi: 10.1007/s00464-014-3687-3.

4. Wang XT, Wang HG, Duan WD, Wu CY, Chen MY, Li H, Huang X, Zhang FB, Dong JH. Pure Laparoscopic Versus Open Liver Resection for Primary Liver Carcinoma in Elderly Patients: A Single-Center, Case-Matched Study. Medicine (Baltimore). 2015; 94: e1854. doi: 10.1097/md.0000000000001854.

5. Kanazawa A, Tsukamoto T, Shimizu S, Kodai S, Yamamoto S, Yamazoe S, Ohira G, Nakajima T. Laparoscopic liver resection for treating recurrent hepatocellular carcinoma. J Hepatobiliary Pancreat Sci. 2013; 20: 512-7. doi: 10.1007/s00534-012-0592-9.

6. Chan AC, Poon RT, Chok KS, Cheung TT, Chan SC, Lo CM. Feasibility of laparoscopic re-resection for patients with recurrent hepatocellular carcinoma. World J Surg. 2014; 38: 1141-6. doi: 10.1007/s00268-013-2380-3.

7. Zhang J, Zhou ZG, Huang ZX, Yang KL, Chen JC, Chen JB, Xu L, Chen MS, Zhang YJ. Prospective, single-center cohort study analyzing the efficacy of complete laparoscopic resection on recurrent hepatocellular carcinoma. Chin J Cancer. 2016; 35: 25. doi: 10.1186/s40880-016-0088-0.

8. Han DH, Choi SH, Park EJ, Kang DR, Choi GH, Choi JS. Surgical outcomes after laparoscopic or robotic liver resection in hepatocellular carcinoma: a propensity-score matched analysis with conventional open liver resection. Int J Med Robot. 2016; 12: 735-42. doi: 10.1002/rcs.1714.

9. Cheung TT, Poon RT, Yuen WK, Chok KS, Jenkins CR, Chan SC, Fan ST, Lo CM. Long-term survival analysis of pure laparoscopic versus open hepatectomy for hepatocellular carcinoma in patients with cirrhosis: a single-center experience. Ann Surg. 2013; 257: 506-11. doi: 10.1097/SLA.0b013e31827b947a.

10. Cheung TT, Poon RT, Dai WC, Chok KS, Chan SC, Lo CM. Pure Laparoscopic Versus Open Left Lateral Sectionectomy for Hepatocellular Carcinoma: A Single-Center Experience. World J Surg. 2016; 40: 198-205. doi: 10.1007/s00268-015-3237-8.

11. Shimada M, Hashizume M, Maehara S, Tsujita E, Rikimaru T, Yamashita Y, Tanaka S, Adachi E, Sugimachi K. Laparoscopic hepatectomy for hepatocellular carcinoma. Surg Endosc. 2001; 15: 541-4. doi: 10.1007/s004640080099.

12. Kaneko H, Takagi S, Otsuka Y, Tsuchiya M, Tamura A, Katagiri T, Maeda T, Shiba T. Laparoscopic liver resection of hepatocellular carcinoma. Am J Surg. 2005; 189: 190-4. doi: 10.1016/j.amjsurg.2004.09.010.

13. Belli G, Limongelli P, Fantini C, D'Agostino A, Cioffi L, Belli A, Russo G. Laparoscopic and open treatment of hepatocellular carcinoma in patients with cirrhosis. Br J Surg. 2009; 96: 1041-8. doi: 10.1002/bjs.6680.

14. Endo Y, Ohta M, Sasaki A, Kai S, Eguchi H, Iwaki K, Shibata K, Kitano S. A comparative study of the long-term outcomes after laparoscopy-assisted and open left lateral hepatectomy for hepatocellular carcinoma. Surg Laparosc Endosc Percutan Tech. 2009; 19: e171-4. doi: 10.1097/SLE.0b013e3181bc4091.

15. Alemi F, Kwon E, Freise C, Kang SM, Hirose R, Stewart L, Corvera CU. Hepatic surgery at a VA tertiary medical center: lessons learned. Am J Surg. 2010; 200: 591-5. doi: 10.1016/j.amjsurg.2010.07.014.

16. Ker CG, Chen JS, Kuo KK, Chuang SC, Wang SJ, Chang WC, Lee KT, Chen HY, Juan CC. Liver Surgery for Hepatocellular Carcinoma: Laparoscopic versus Open Approach. Int J Hepatol. 2011; 2011: 596792. doi: 10.4061/2011/596792.

17. Nguyen KT, Marsh JW, Tsung A, Steel JJ, Gamblin TC, Geller DA. Comparative benefits of laparoscopic vs open hepatic resection: a critical appraisal. Arch Surg. 2011; 146: 348-56. doi: 10.1001/archsurg.2010.248.

18. Ai JH, Li JW, Chen J, Bie P, Wang SG, Zheng SG. Feasibility and safety of laparoscopic liver resection for hepatocellular carcinoma with a tumor size of 5-10 cm. PLoS One. 2013; 8: e72328. doi: 10.1371/journal.pone.0072328.

19. Kanazawa A, Tsukamoto T, Shimizu S, Kodai S, Yamazoe S, Yamamoto S, Kubo S. Impact of laparoscopic liver resection for hepatocellular carcinoma with F4-liver cirrhosis. Surg Endosc. 2013; 27: 2592-7. doi: 10.1007/s00464-013-2795-9.

20. de'Angelis N, Memeo R, Calderaro J, Felli E, Salloum C, Compagnon P, Luciani A, Laurent A, Cherqui D, Azoulay D. Open and laparoscopic resection of hepatocellular adenoma: trends over 23 years at a specialist hepatobiliary unit. HPB (Oxford). 2014; 16: 783-8. doi: 10.1111/hpb.12257.

21. Kamiyama T, Tahara M, Nakanishi K, Yokoo H, Kamachi H, Kakisaka T, Tsuruga Y, Matsushita M, Todo S. Long-term outcome of laparoscopic hepatectomy in patients with hepatocellular carcinoma. Hepatogastroenterology. 2014; 61: 405-9. doi:

22. Yamashita Y, Ikeda T, Kurihara T, Yoshida Y, Takeishi K, Itoh S, Harimoto N, Kawanaka H, Shirabe K, Maehara Y. Long-term favorable surgical results of laparoscopic hepatic resection for hepatocellular carcinoma in patients with cirrhosis: a single-center experience over a 10-year period. J Am Coll Surg. 2014; 219: 1117-23. doi: 10.1016/j.jamcollsurg.2014.09.003.

23. Cho JY, Han HS, Yoon YS, Choi Y, Lee W. Outcomes of laparoscopic right posterior sectionectomy in patients with hepatocellular carcinoma in the era of laparoscopic surgery. Surgery. 2015; 158: 135-41. doi: 10.1016/j.surg.2015.02.007.

24. Harimoto N, Ikeda T, Takeishi K, Itoh S, Yamashita Y, Ikegami T, Yoshizumi T, Kawanaka H, Shirabe K, Maehara Y. Outcomes After Laparoscopic Hepatectomy in the Semi-prone Position for Hepatocellular Carcinoma Located in Segment 6, 7, or 8. Anticancer Res. 2015; 35: 4167-70. doi:

25. Li W, Zhou X, Huang Z, Zhang H, Zhang L, Shang C, Chen Y. Laparoscopic surgery minimizes the release of circulating tumor cells compared to open surgery for hepatocellular carcinoma. Surg Endosc. 2015; 29: 3146-53. doi: 10.1007/s00464-014-4041-5.

26. Martin RC, 2nd, Mbah NA, St Hill R, Kooby D, Weber S, Scoggins CR, Maithel SK. Laparoscopic versus open hepatic resection for hepatocellular carcinoma: improvement in outcomes and similar cost. World J Surg. 2015; 39: 1519-26. doi: 10.1007/s00268-015-2974-z.

27. Xiao L, Xiang LJ, Li JW, Chen J, Fan YD, Zheng SG. Laparoscopic versus open liver resection for hepatocellular carcinoma in posterosuperior segments. Surg Endosc. 2015; 29: 2994-3001. doi: 10.1007/s00464-015-4214-x.

28. Ahn S, Cho A, Kim EK, Paik KY. Favorable Long-Term Oncologic Outcomes of Hepatocellular Carcinoma Following Laparoscopic Liver Resection. J Laparoendosc Adv Surg Tech A. 2016; 26: 447-52. doi: 10.1089/lap.2015.0534.

29. Harada N, Maeda T, Yoshizumi T, Ikeda T, Kayashima H, Ikegami T, Harimoto N, Takaki S, Maehara Y. Laparoscopic Liver Resection Is a Feasible Treatment for Patients with Hepatocellular Carcinoma and Portal Hypertension. Anticancer Res. 2016; 36: 3489-97. doi:

30. Lai C, Jin RA, Liang X, Cai XJ. Comparison of laparoscopic hepatectomy, percutaneous radiofrequency ablation and open hepatectomy in the treatment of small hepatocellular carcinoma. J Zhejiang Univ Sci B. 2016; 17: 236-46. doi: 10.1631/jzus.B1500322.

31. Xiang L, Li J, Chen J, Wang X, Guo P, Fan Y, Zheng S. Prospective cohort study of laparoscopic and open hepatectomy for hepatocellular carcinoma. Br J Surg. 2016; 103: 1895-901. doi: 10.1002/bjs.10294.

32. Zhang Y, Chen XM, Sun DL. Short-term Outcomes of Laparoscopic Versus Open Right Hemihepatectomy for Hepatocellular Carcinoma. Surg Laparosc Endosc Percutan Tech. 2016; 26: e157-e60. doi: 10.1097/sle.0000000000000355.

33. Zhang Y, Huang J, Chen XM, Sun DL. A Comparison of Laparoscopic Versus Open Left Hemihepatectomy for Hepatocellular Carcinoma. Surg Laparosc Endosc Percutan Tech. 2016; 26: 146-9. doi: 10.1097/sle.0000000000000247.

34. Amato B, Aprea G, De Rosa D, Milone M, di Domenico L, Amato M, Compagna R, Santoro M, Johnson LB, Sanguinetti A, Polistena A, Avenia N. Laparoscopic hepatectomy for HCC in elderly patients: risks and feasibility. Aging Clin Exp Res. 2017; 29: 179-83. doi: 10.1007/s40520-016-0675-6.

35. Chen J, Li H, Liu F, Li B, Wei Y. Surgical outcomes of laparoscopic versus open liver resection for hepatocellular carcinoma for various resection extent. Medicine (Baltimore). 2017; 96: e6460. doi: 10.1097/md.0000000000006460.

36. Guro H, Cho JY, Han HS, Yoon YS, Choi Y, Jang JS, Kwon SU, Kim S, Choi JK. Laparoscopic liver resection of hepatocellular carcinoma located in segments 7 or 8. Surg Endosc. 2017. doi: 10.1007/s00464-017-5756-x.

37. Ryu T, Honda G, Kurata M, Kobayashi S, Sakamoto K, Honjo M. Perioperative and oncological outcomes of laparoscopic anatomical hepatectomy for hepatocellular carcinoma introduced gradually in a single center. Surg Endosc. 2017. doi: 10.1007/s00464-017-5745-0.

38. Laurent A, Cherqui D, Lesurtel M, Brunetti F, Tayar C, Fagniez PL. Laparoscopic liver resection for subcapsular hepatocellular carcinoma complicating chronic liver disease. Arch Surg. 2003; 138: 763-9; discussion 9. doi: 10.1001/archsurg.138.7.763.

39. Lai EC, Tang CN, Ha JP, Li MK. Laparoscopic liver resection for hepatocellular carcinoma: ten-year experience in a single center. Arch Surg. 2009; 144: 143-7; discussion 8. doi: 10.1001/archsurg.2008.536.

40. Sarpel U, Hefti MM, Wisnievsky JP, Roayaie S, Schwartz ME, Labow DM. Outcome for patients treated with laparoscopic versus open resection of hepatocellular carcinoma: case-matched analysis. Ann Surg Oncol. 2009; 16: 1572-7. doi: 10.1245/s10434-009-0414-8.

41. Aldrighetti L, Guzzetti E, Pulitano C, Cipriani F, Catena M, Paganelli M, Ferla G. Case-matched analysis of totally laparoscopic versus open liver resection for HCC: short and middle term results. J Surg Oncol. 2010; 102: 82-6. doi: 10.1002/jso.21541.

42. Hu BS, Chen K, Tan HM, Ding XM, Tan JW. Comparison of laparoscopic vs open liver lobectomy (segmentectomy) for hepatocellular carcinoma. World J Gastroenterol. 2011; 17: 4725-8. doi: 10.3748/wjg.v17.i42.4725.

43. Meguro M, Mizuguchi T, Kawamoto M, Ota S, Ishii M, Nishidate T, Okita K, Kimura Y, Hirata K. Clinical comparison of laparoscopic and open liver resection after propensity matching selection. Surgery. 2015; 158: 573-87. doi: 10.1016/j.surg.2015.02.031.
